# Mosaic trisomy 8 detected by fibroblasts cultured of skin

**Published:** 2016-06-30

**Authors:** Gustavo Giraldo, Ana M Gómez, Lina Mora, Fernando Suarez-Obando, Olga Moreno

**Affiliations:** 1 Clínica Universitaria Bolivariana, Universidad Pontificia Bolivariana, Medellín, Colombia; 2 Instituto de Genética Humana, Facultad de Medicina, Pontificia Universidad Javeriana, Bogotá, Colombia; 3 Hospital Universitario San Ignacio, Bogotá, Colombia

**Keywords:** Mosaicism, Chromosome 8, mosaic trisomy, Skin Abnormalities, Intellectual disability

## Abstract

**Introduction:**

Mosaic trisomy 8 or "Warkany's Syndrome" is a chromosomopathy with an estimated prevalance of 1:25,000 to 1:50,000, whose clinical presentation has a wide phenotypic variability.

**Case Description:**

Patient aged 14 years old with antecedents of global retardation of development, moderate cognitive deficit and hypothyroidism of possible congenital origin.

**Clinical Findings:**

Physical examination revealed palpebral ptosis, small corneas and corectopia, hypoplasia of the upper maxilla and prognathism, dental crowding, high-arched palate, anomalies of the extremities such as digitalization of the thumbs, clinodactyly and bilateral shortening of the fifth finger, shortening of the right femur, columnar deviation and linear brown blotches that followed Blaschko's lines. Cerebral nuclear magnetic resonance revealed type 1 Chiari's malformation and ventriculomegaly. Although the karyotype was normal in peripheral blood (46,XY), based on the finding of cutaneous mosaicism the lesions were biopsied and cytogenetic analysis demonstrated mosaic trisomy 8: mos 47,XY,+8[7]/46,XY[93].

**Clinical Relevance:**

Trisomy 8 is clinically presented as a mosaic, universal cases being unfailingly lethal. In this particular case, cutaneous lesions identified the mosaic in tissue, although the karyotype was normal in peripheral blood. The cutaneous mosaicism represented by brown linear blotches which follow Blaschko's lines is a clinical finding that has not previously been described in Warkany's syndrome.

## Abstract (es)

**Introducción:**

La trisomía 8 en mosaico o Síndrome de Warkany, es una cromosomopatía con una prevalencia estimada de 1:25,000 a 1:50,000, que se presenta clínicamente con una amplia variabilidad fenotípica.

**Descripción del Caso:**

Paciente de 14 años con antecedente de retardo global del desarrollo, déficit cognitivo moderado e hipotiroidismo de posible origen congénito.

**Hallazgos Clínicos:**

Al examen físico presenta ptosis palpebral, corneas pequeñas y corectopia, hipoplasia de maxilar superior y prognatismo, apiñamiento dental, paladar alto ojival, anomalías en extremidades como digitalización de pulgares, clinodactilia y acortamiento bilateral del quinto dedo en manos, acortamiento de fémur derecho, desviación de columna y máculas lineales pardas que siguen las líneas de Blaschko. En la resonancia nuclear magnética cerebral se aprecia malformación de Chiari tipo 1 y ventriculomegalia. El cariotipo en sangre periférica fue normal (46,XY) sin embargo, ante el hallazgo de mosaicismo cutáneo, se realizó biopsia de las lesiones y su análisis citogenético demostró trisomía 8 en mosaico: mos47,XY,+8[7] /46,XY[93].

**Relevancia Clínica:**

La trisomía 8 se presenta clínicamente en mosaico, los casos universales son indefectiblemente letales. En este caso particular, las lesiones cutáneas identificaron el mosaico en tejido, frente al cariotipo normal en sangre periférica. El mosaicismo cutáneo representado por las máculas lineales pardas (que siguen las líneas de Blaschko) es un hallazgo clínico que no se había descrito en el síndrome de Warkany

## Introduction

Mosaic trisomy 8 or Warkany's syndrome is a chromosomal anomaly with an estimated prevalence of 1:25,000 to 1:50,000 1 and male/female sex ratio of 5:1 2. Universal trisomy 8 is lethal and accounts for 0.7%-0.8% of spontaneous abortions 3. This syndrome has a wide phenotypic variability, including mild to severe intellectual disability, deficit in growth, craneo-facial dysmorphism, skeletal anomalies (principally vertebral and costal alterations), diminished articular mobility, camptodactyly, cardiac alterations and agenesia of the corpus callosum. Deep furrows on the soles of the feet are highly characteristic 4. Diagnosis of trisomy 8 is based on the presence of an extra chromosome 8 in mosaic, associated with the normal cell line. The distribution of the extra chromosome varies from one patient to another and between tissues. In certain cases, chromosomal alteration is only found in fibroblasts, while in other patients it may predominate in the lymphocytes and not manifest itself in other tissues or appear in only a small proportion of those affected. Patients have been described with complete trisomy in lymphocytes and mosaicism present in at least one of the other tissues examined 5.

There does not appear to be a correlation between the proportion of trisomic cells and the severity of clinical manifestations. Furthermore, trisomy occurs *de novo* given that carrier parents have not been identified. Its cause may be meiotic nondisjunction (pre-zygotic) with partial post-zygotic loss of the extra chromosome 8 or post-zygotic mitotic nondisjunction. Mitotic nondisjunction appears to be more frequent, which would explain the mosaicism, lengthy survival and good clinical prognosis of these patients 5-8 .

The following report describes the case of a patient with mosaic trisomy 8, with a varied clinical presentation associated with cutaneous mosaicism. A diagnosis of mosaic trisomy 8 was made based on the karyotype of fibroblasts, that of peripheral blood being normal. 

## Case Description

**Figure 1:**
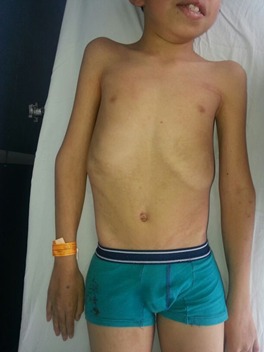
Frontal view of patient in which linear blotches (in the thorax and abdomen) and alterations of the extremities can be observed (digitalization of the thumbs and shortening of the fifth finger of the right hand).

**Figure 2.**
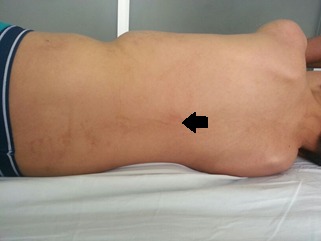
Dorsal view of patient showing brown linear blotches that followed Blaschko's lines.

The patient was a 14-year-old from Colombia, the son of parents without important medical antecedents, who were not blood relatives and were both aged 26 years in the moment of birth. He was the product of a fifth pregnancy, which it was not controlled or had prenatal screening, born by caesarian section at 40 weeks' gestation after premature rupture of the membranes. The patient had shown global retardation of development, indicated by his not being able to hold his head up until he was three years old, sit up until five or walk unaided before he was six. He also showed retardation in speech development. The developmental milestones were attained after intervention with physiotherapy. In addition, the patient had antecedents of hypothyroidism diagnosed at the age of nine years old, although with no other apparent causes this was presumed to be of congenital origin and was being managed with levothyroxine. At the time of the study, the patient had moderate cognitive deficit. Physical examination revealed facial defects such as palpebral ptosis, small corneas and corectopia, hypoplasia of the upper maxilla and prognathism, dental crowding, high-arched palate, anomalies of the extremities such as digitalization of the thumbs, clinodactyly and bilateral shortening of the fifth finger, deep furrows in the palms and soles of the feet, shortening of the right femur and scoliosis. The skin presented brown linear blotches that followed Blaschko's lines which may reflect an embryological development (Fig. 1 and 2). We didn't have access to histological results of biopsy of skin.

**Figure 3.**
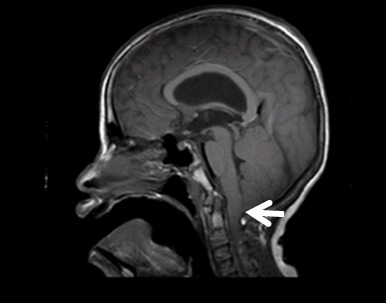
Cerebral nuclear magnetic resonance of the patient that show increased ventricular system without signs of edema and the arrow show the herniated cerebellum.

Cerebral nuclear magnetic resonance revealed type 1 Chiari's malformation and ventriculomegaly (Fig. 3).

**Figure 4.**
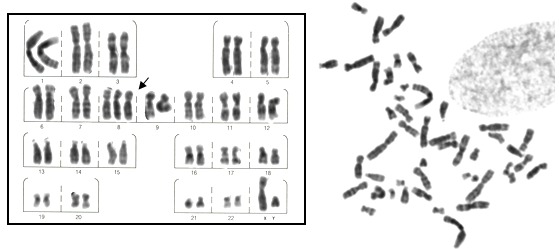
G-banding karyotype with 8 trisomy (arrows) of fibroblasts cultivated from biopsy of the cutaneous lesions final karyotype mos 47,XY,+8[7]/46,XY[93].

Karyotyping of peripheral blood at G-banding (100 metaphases) gave a normal result of 46,XY. The karyotype of fibroblasts in skin without cutaneous lesions showed a result of 46,XY in 100 G-banding metaphases analyzed. On finding cutaneous mosaicism a biopsy of the blotches was carried out and the karyotype of fibroblasts analysed, revealing a low frequency mosaicism of chromosome 8 trisomy: mos 47,XY,+8[7]/46,XY[93] (Fig. 4). 

### Ethical considerations

The legal guardian of the patient signed the informed consent form allowing photographs to be taken and/or audiovisual recordings of medical genetics to be made, thus authorizing the images to be used in medical publications, including articles, books and electronic publications on the understanding that these could be viewed by members of the general public, scientists and medical researchers who regularly use these publications in their professional development.

## Discussion

Trisomy 8 or Warkany's syndrome tends to be presented as a mosaic, cases being universally lethal in the first months of life. In this case, the two cell lines were encountered in fibroblasts, while lymphocytes of peripheral blood only showed the normal chromosomal complement. Warkany's syndrome presents a wide phenotypic variability, as observed in a series of reported cases, in both the clinical presentation and cytogenetic analyses are variable. However, a common characteristic of these patients is the presence of deep furrows in the palms and soles of the feet. In the Table 1 we can observe the vast variability of the phenotype between the different patients with this condition, but we can conclude that the alterations in extremities, deep furrows in palms or soles of feet and global retardation of development are the common characteristics found in patients with Warkany's syndrome, which are in our patient. In the present case, chromosomal mosaicism was suspected based on the presence of a cutaneous mosaicism represented by brown linear blotches that followed Blaschko's lines, that represent a nonrandom developmental pattern of the skin, fundamentally differing from the system of dermatomes. A wide range of skin disorders, both congenital and acquired, can be distributed along Blaschko's lines, which are thought to represent the pathways of embryonic cell development 9.

**Table 1.** Phenotypic description and cytogenetic status of cases of trisomy 8 in mosaic reported in the literature compared with the patient reported here.

Reference	1	4	4	4	4	10	11	12	12	13	14	15	16	Our patient
Case		1	2	3	4			1	2					
Alterations														
Prominent forehead	-	-	-	+	-	+	+	+	+	+	-	-	-	-
Broad nose	-	+	+	+	+	+	-	+	-	+	+	-	-	-
Alterations in PA	-	+	+	+	+	+	-	+	-	-	+	-	-	-
Sunken eyes	-	+	+	-	+	+	-	-	+	-	-	-	-	-
Everted/thick lips	+	+	+	+	-	+	+	+	-	+	-	-	-	-
Arched palate	-	-	+	-	+	+	+	-	-	+	-	-	+	+
Palate fissure	-	-	-	+	-	-	+	-	-	-	+	-	-	-
Short neck	-	+	+	-	-	-	-	+	-	-	-	-	-	-
Campodactyly of fingers	+	+	+	-	-	+	-	+	-	-	-	+	-	+
Deep furrows in palms or soles of feet	+	+	+	+	+	+	+	-	+	+	-	+	-	+
Alterations in extremities	+	+	+	+	+	+	+	+	+	+	+	+	-	+
Pectus excavatum (hollowed chest)	-	+	-	-	-	-	-	-	+	-	-	-	-	-
Vertebral anomalies	-	-	-	-	-	-	+	-	+	-	-	-	+	+
Congenital cardiopathy	-	-	-	-	+	-	-	+	-	-	-	+	-	-
Urogenital anomalies	+	-	+	-	-	-	+	+	-	+	-	+	+	-
Global retardation of development	+	+	+	+	-	+	+	+	-	+	-	-	+	+
Skin anomalies	-	-	-	-	-	-	+	-	-	-	+	-	-	+
Karyotype	mos47,XY,+8[7[/46,XY[43[	mos47,XY,+8[28[/ 46,XY[2[	mos47,XY,+8[3[/46.XY[27[	mos47,XY,+8 [18[/46,XY [89[	mos47,XY,+8[40[/46.XY[60[	mos47,XY,+8[15[/46.XY[15[	mos47,XY,+8[90[/46.XY[10[	mos47,XY,+8[74[/46,XY[26[	mos47,XX,+8[94[/46,XX[6[	mos47,XY,+8[21[/46,XY[24[	mos47,XX,8[17[/46,XX[83[	mos47,XY,+8[29[/46,XY[21[	mos46,XY/47,XY,(+8)(8p21.2→8q12.1)	mos47,XY,+8[7[/46,XY[93[

Although some neoplasms have been reported in Warkany's syndrome, mainly hematologic malignancies (that might be related to the finding of trisomy 8 at the somatic level in some of them such as chronic myeloid leukemia or myelodysplastic syndromes), to the best of our knowledge there are not reported cases of malignant transformation of cutaneous lesions found in cases of cutaneous mosaicism.

Mosaicism presented only in fibroblasts has been reported previously. It has also been reported that trisomic cells in lymphocytes may disappear with age, due to the selective advantage of normal cells. This can be explained by variation in the cellular growth rates, delayed anaphase, delayed entry into the G_0_ phase or apoptosis, as mentioned by Hulley *et al* 5 *,* 17. This mechanism could in turn explain why lymphocytes showed a normal karyotype in the case presented here. 

Pigmentary anomalies of skin have been described in relation to chromosomal mosaicism, generally in patients who also present cognitive compromise and certain dismorphic characteristics 18, such as the present case. Brown blotches that follow Blaschko's lines also have been described in mosaics related to alterations in sexual chromosomes and autosomes (trisomy 7, 9, 13, 14, 18, among others), as well as diverse structural alterations 19,20. However this is the first case in which a mosaical trisomy of chromosome 8 is described, identified from karyotyping of brown blotches in the skin biopsy and demonstrating the need to carry out cytogenetic studies in patients with anomalies associated with cutaneous mosaicism.

## Conclusion 

Mosaic trisomy of chromosome 8 or "Warkany's syndrome" has a wide phenotypic variability and cutaneous lesions have been reported in some cases. It is concluded that patients with pigmentary anomalies (principally when these follow Blaschko's lines) should be evaluated to search for chromosomal anomalies in mosaic, requiring cytogenetic studies to be carried out on different tissues on repeated occasions. 
